# Diagnostic value of an algorithm for autoimmune epilepsy in a retrospective cohort

**DOI:** 10.3389/fneur.2022.902157

**Published:** 2022-09-14

**Authors:** Mitsuhiro Sakamoto, Riki Matsumoto, Akihiro Shimotake, Jumpei Togawa, Hirofumi Takeyama, Katsuya Kobayashi, Frank Leypoldt, Klaus-Peter Wandinger, Takayuki Kondo, Ryosuke Takahashi, Akio Ikeda

**Affiliations:** ^1^Department of Neurology, Kyoto University Graduate School of Medicine, Kyoto, Japan; ^2^Department of Neurology, Rakuwakai Otowa Hospital, Kyoto, Japan; ^3^Division of Neurology, Kobe University Graduate School of Medicine, Kobe, Japan; ^4^Department of Respiratory Care and Sleep Control Medicine, Kyoto University Graduate School of Medicine, Kyoto, Japan; ^5^Department of Neurology, Japanese Red Cross Otsu Hospital, Otsu, Japan; ^6^Neuroimmunology, Institute of Clinical Chemistry, University Hospital Schleswig-Holstein, Kiel, Germany; ^7^Neuroimmunology, Institute of Clinical Chemistry, University Hospital Schleswig-Holstein, Lübeck, Germany; ^8^Department of Neurology, Kansai Medical University Medical Center, Moriguchi, Japan; ^9^Department of Epilepsy, Movement Disorders and Physiology, Kyoto University Graduate School of Medicine, Kyoto, Japan

**Keywords:** observational study, epilepsy, focal seizures, autoimmune disease, diagnostic test assessment

## Abstract

**Purpose:**

This study aims to propose a diagnostic algorithm for autoimmune epilepsy in a retrospective cohort and investigate its clinical utility.

**Methods:**

We reviewed 60 patients with focal epilepsy with a suspected autoimmune etiology according to board-certified neurologists and epileptologists. To assess the involvement of the autoimmune etiology, we used the patients' sera or cerebrospinal fluid (CSF) samples to screen for antineuronal antibodies using rat brain immunohistochemistry. Positive samples were analyzed for known antineuronal antibodies. The algorithm applied to assess the data of all patients consisted of two steps: evaluation of clinical features suggesting autoimmune epilepsy and evaluation using laboratory and imaging findings (abnormal CSF findings, hypermetabolism on fluorodeoxyglucose-positron emission tomography, magnetic resonance imaging abnormalities, and bilateral epileptiform discharges on electroencephalography). Patients were screened during the first step and classified into five groups according to the number of abnormal laboratory findings. The significant cutoff point of the algorithm was assessed using a receiver-operating characteristic curve analysis.

**Results:**

Fourteen of the 60 patients (23.3%) were seropositive for antineuronal antibodies using rat brain immunohistochemistry. Ten patients had antibodies related to autoimmune epilepsy/encephalitis. The cutoff analysis of the number of abnormal laboratory and imaging findings showed that the best cutoff point was two abnormal findings, which yielded a sensitivity of 78.6%, a specificity of 76.1%, and an area under the curve of 0.81.

**Conclusion:**

The proposed algorithm could help predict the underlying autoimmune etiology of epilepsy before antineuronal antibody test results are available.

## Introduction

As a result of advancements in antibody detection technology since the 2000s, antibodies against antineuronal cell-surface antigens have been discovered (1, 2), and many types of autoimmune encephalitis associated with these antibodies have been reported. The existence of epilepsy syndrome associated with an autoimmune etiology was proposed in the early twenty-first century (3), and it has also been demonstrated that antineuronal antibodies are present in patients with classical focal epilepsy syndromes (4). In the latest International League Against Epilepsy (ILAE) classification of epilepsy in 2017, immunity was adopted for the first time as one of the etiologies of epilepsy (5). It has been reported that immunotherapy is successful in treating antineuronal antibody-positive refractory epilepsy and that early treatment improves the patient prognosis (6, 7). However, in real-world medical practice, antibody testing may be inaccessible, thereby rendering an early diagnosis of autoimmune epilepsy difficult worldwide. Additionally, patients may have epilepsy associated with an autoimmune etiology despite negative antineuronal antibody results. Recently, clinical features suggestive of autoimmune epilepsy have been proposed (7), and a clinical approach to the diagnosis of autoimmune encephalitis has been presented (8). The antibody prevalence in epilepsy (APE) score and the antibodies contributing to focal epilepsy signs and symptoms (ACES) score may be helpful when selecting patients who require antibody testing (9, 10). However, definitive diagnostic criteria have not been established. Herein, we propose and validate a diagnostic algorithm for autoimmune epilepsy in a cohort of patients who underwent antineuronal antibody testing. We adopted this algorithm approach without antibody testing because this follows the real-life medical process that ranges from assessing the clinical history and symptoms to laboratory examinations.

## Materials and methods

### Standard protocol approvals, registrations, and patient consent

This study was approved by the ethics committee of the Kyoto University Graduate School of Medicine (no. C-0588). All patients provided written informed consent.

The cohort consisted of 60 patients with focal epilepsy suspected of having an autoimmune etiology according to board-certified neurologists and epileptologists at the clinic of Kyoto University Hospital. All patients were admitted to the hospital from January 2012 to March 2017, and underwent comprehensive evaluations for epilepsy. We included 40 out of 70 patients from the preliminary cohort (11) whose serum or CSF samples were available to check the rat brain immunohistochemistry and comprehensive antibody testing, and added 20 patients who were admitted after the previous study had been completed. When patients had other obvious neurological diseases, such as paraneoplastic neurological syndrome and multiple sclerosis (MS), they were excluded from the present study. Two patients were excluded. The first patient was diagnosed as having paraneoplastic neurological syndrome because of typical symptoms, and he suddenly died from advanced gastric cancer after being diagnosed. The second one was diagnosed with tumefactive MS in light of the MRI findings.

### Immunological analysis

The cerebrospinal fluid (CSF) or serum samples were initially screened for reactivity with rat brain through immunohistochemistry as reported elsewhere (12, 13). In brief, fresh rat brains were split, briefly fixed in 4°C paraformaldehyde in PBS for 30 min, and then dehydrated in 40% sucrose in PBS at 4°C overnight. Brains were frozen in liquid nitrogen and cut into 7 μm sections on a cryostat and transferred onto coverslips. After thawing, coverslips were washed in PBS, treated with 0.3% H_2_O_2_ in PBS, and blocked with 5% goat serum followed by incubation with serum at 1:200 or CSF at 1:4 in blocking solution overnight at 4°C. After washing, coverslips were then incubated with a goat anti-human IgG (H+L) biotinylated antibody (Vector, BA-3000) followed by staining with ABC Elite Kit (Vector PK6100) according to the manufacturer's instructions. Sections were analyzed using a Zeiss Axioscope by at least two experienced investigators blinded to the experimental conditions. Positive samples were further investigated for reactivity against specific known antigens in a sequential manner (14). First, we investigated the levels of proteins related to autoimmune epilepsy/limbic encephalitis using standardized commercially available test kits (immunofluorescence tests with tissue and fixed transfected cells, enzyme-linked immunosorbent assay, and immunoblotting [EUROIMMUN, Lubeck, Germany]). The following proteins were targeted: N-methyl-D-aspartate receptor (NMDAR); α-amino-3-hydroxy-5-methyl-4-isoxazole propionic acid receptor (AMPAR); LGI1; contactin-associated protein-like 2 (CASPR2); Delta/Notch-like epidermal growth factor-related receptor (DNER); Zic4; dipeptidyl-peptidase-like protein 6 (DPPX); γ-aminobutyric acid type B receptor (GABABR); Hu, Yo, Ri, Ma, and collapsin response mediator protein 5 (CRMP-5/CV2); and amphiphysin. Immunoreactivity for GAD65 was tested using an enzyme-linked immunosorbent assay and immunofluorescence with tissue sections. Thereafter, the negative samples were tested for the presence of antibodies against rarely occurring antigens [that is, metabotropic glutamate receptor 1 and metabotropic glutamate receptor 5 (mGluR1 and mGluR5)] (15, 16), γ-aminobutyric acid type A receptor (GABAAR), and IgLON 5 (17, 18) using specifically transfected cells. Additionally, we assessed the myelin-oligodendrocyte glycoprotein (MOG) titers with a full-length human MOG construct transfected into HEK293T cells using indirect immunofluorescence on live, non-permeabilized cells for 12 patients whose serum was available (19). All samples that tested positive with cell-based assays, either commercial or in-house, were characterized using end-point dilutions.

We reported samples as positive if they had staining of hippocampal neuropil or GAD-typical staining (as positive GAD). We considered the nuclear stains sample to be positive, but there was no sample in this study. We considered the patients with antibodies to detectable known target antigens, that is, known antineuronal antibodies, to have “definite autoimmune epilepsy.” Patients whose CSF samples showed positive reactivity in rat brain immunohistochemistry but were negative for known antineuronal antibodies were considered to have “probable seropositive autoimmune epilepsy.” When the serum samples had positive rat brain immunohistochemistry but the CSF samples were negative, or when CSF samples were not available, we considered these patients to have “possible seropositive autoimmune epilepsy.”

Thereafter, we retrospectively applied the diagnostic algorithm to our cohort. To analyze our algorithm, we considered patients with seropositivity for antineuronal antibodies according to an assessment using rat brain immunohistochemistry (herein referred to as seropositive) to have autoimmune epilepsy with a definite, probable, or possible seropositive status and validated the usefulness of the diagnostic algorithm.

### Algorithm

**Figure 1:**
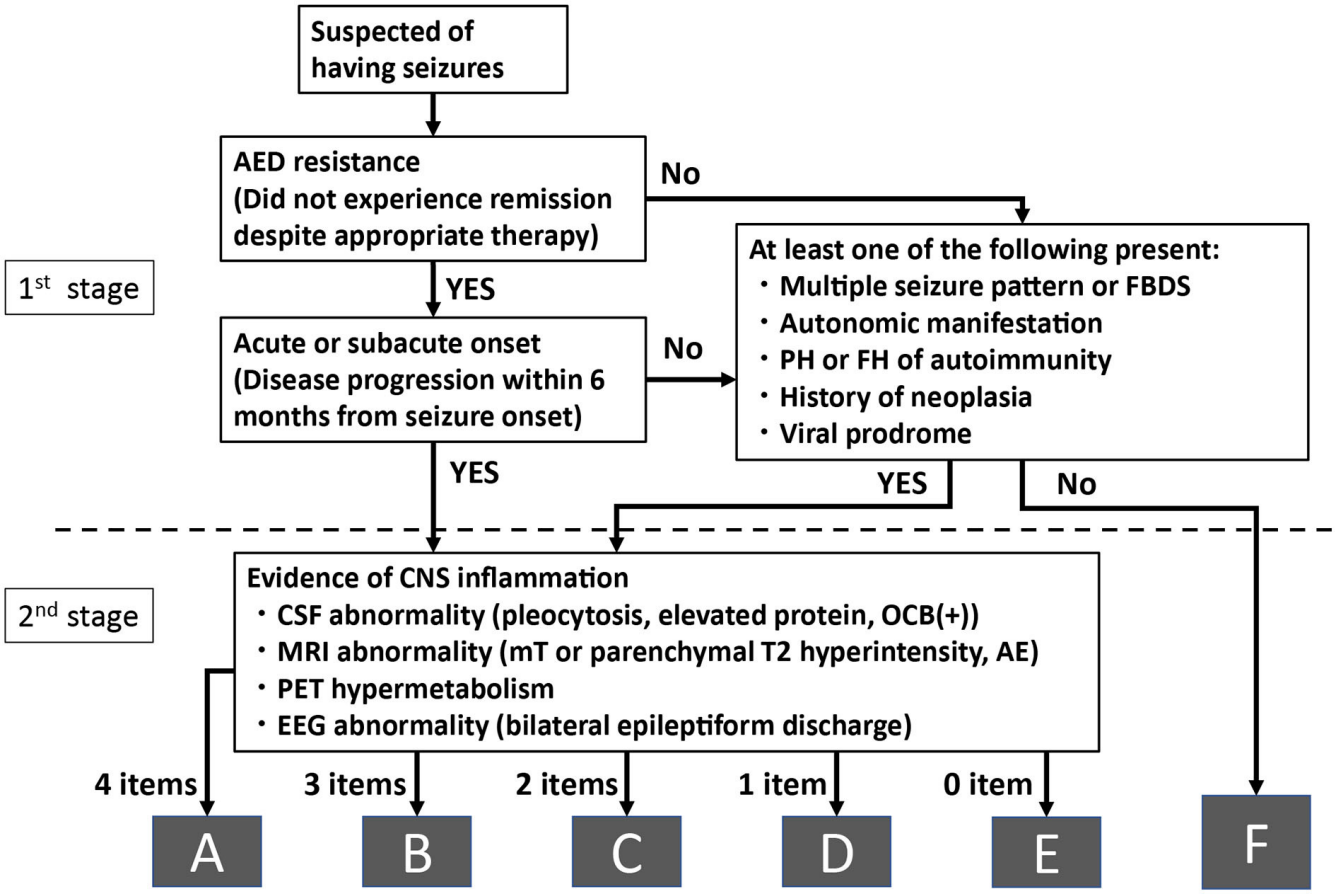
Algorithm for diagnosing autoimmune epilepsy without evaluating antineuronal antibodies. AE, amygdala enlargement; AED, antiepileptic drugs; CNS, central nervous system; CSF, cerebrospinal fluid; EEG, electroencephalography; FBDS, faciobrachial dystonic seizure; FH, family history; MRI, magnetic resonance imaging; mT, medial temporal; OCB, oligoclonal bands; PET, positron emission tomography; PH, past history.

In a real-world clinical environment, the medical history is first assessed, followed by laboratory tests, evaluation of these test results, and the final diagnosis. We previously proposed such a diagnostic algorithm for autoimmune epilepsy and evaluated its clinical utility (11). In this preliminary investigation, the diagnostic algorithm followed the “real-world” practical procedures, from assessing medical history/clinical symptoms to laboratory examinations, taking into account the “clinical features suggesting autoimmune epilepsy” (7). Based on our preliminary investigation, which included several false-negative results, we revised the diagnostic algorithm by adding peri-ictal autonomic symptoms and electroencephalography (EEG) findings and evaluated its utility in a larger cohort who underwent comprehensive antineuronal antibody testing (Figure 1) (8, 20, 21). The algorithm consisted of two stages. During the first stage, we assessed the clinical information, such as the medical history and ictal symptoms. Specifically, we assessed “refractory seizure to appropriate drug therapy” and “acute or subacute onset (disease progression within 6 months from seizure onset)” or having one of the following characteristic clinical features: “multiple seizure types or facial brachial dystonic seizures (FBDS),” “ictal autonomic manifestations,” “past or family history of autoimmune disease,” “history of malignancy,” and “virus prodrome” (22). Regarding the ictal autonomic manifestations, we included cardiovascular, respiratory, gastrointestinal, thermoregulatory, vasomotor, pilomotor, sensory, genital, and urinary manifestations; however, we excluded epigastric rising sensation, nausea, and urinary incontinence because these symptoms are commonly observed with typical mesial temporal lobe epilepsy, such as hippocampal sclerosis.

When applying this algorithm to the retrospective cohort, we considered the patients to have acute or subacute onset when they were admitted to the hospital for evaluation and treated within 6 months of their seizure onset.

When the patients satisfied any of these conditions, we considered that they had a high possibility of autoimmune epilepsy based on clinical reasons and recommended that they proceed to the second stage for further laboratory analyses. When the patients did not satisfy the first stage of the algorithm, they were classified into the group with a low possibility of autoimmune epilepsy (group F).

During the second stage, we evaluated the results of four examinations listed below:

1) “Abnormal CSF finding:” elevated cell count (>5 cells per μL), elevated protein levels (> 40 mg / dl), or positive oligoclonal band.
2) “Abnormal brain magnetic resonance imaging (MRI) findings” (medial temporal or parenchymal including white matter lesions hyperintense signal on T2-weighted image (T2WI) or fluid-attenuated inversion recovery sequence (FLAIR) and/or amygdala enlargement).
3) “Hypermetabolism on fluorodeoxyglucose-positron emission tomography (FDG-PET).”
4) “EEG with bilateral epileptiform discharge (independently recorded either ictally or interictally).”

Patients who had positive findings in all the four examinations during the second stage were classified into group A. Similarly, when patients had three, two, one, or none of the positive findings in four examinations during the second stage, they were classified into groups B, C, D, and E, respectively.

Abnormal MRI and FDG-PET findings were determined by visual inspection. Amygdala enlargement was defined as a unilateral or bilateral enlargement of the amygdala on FLAIR or T2WI, irrespective of signal changes. The findings were judged by three board-certified neurologists or radiologists to reach a consensus. We considered the findings significant when two or more evaluators observed abnormal findings. We only evaluated the MRI results obtained at our hospital. We included bilateral epileptiform discharges and EEG seizure patterns (including periodic patterns) as abnormal EEG findings in this article, and we did not mention the specific EEG findings for autoimmune encephalitis, such as extreme delta brush.

### Comparison with other scores

To compare the diagnostic value of the algorithm with that of the previously published APE score (9) and ACES score (10), we applied these scores to the patients in our cohort and calculated the sensitivity and specificity of each score. We then compared the diagnostic value of our algorithm with that of the scores.

### Statistical analysis

Univariate analyses of nominal and interval variables were performed using the Fisher exact test and the Wilcoxon test, respectively. To evaluate this diagnosis algorithm, we conducted a receiver operating characteristic (ROC) curve analysis (23). The optimal cutoff was determined by “the closest to (0, 1) criteria”: the point on the curve closest to the point which implies the perfect scenario, 100% sensitivity, and 100% specificity.

## Results

The algorithm was applied to 60 patients. Thirty-four patients (57%) were female and the median age at onset was 55 years (range: 9–83 years) (Table 1). MRI and EEG were performed for all patients, but the CSF and FDG-PET examinations were performed for 55 and 58 patients, respectively (Table 2). Fourteen patients had CSF or serum reactivity with rat brain immunohistochemistry [CSF: 9/49 patients (18%); serum: 5/11 patients (45%)]. Among 14 patients with positive immunohistochemistry results, monospecific cell-based assays against the specific antigens related to autoimmune epilepsy/limbic encephalitis revealed that 10 patients had antibodies against a specific antigen (LGI1, 5 patients; GAD, 4 patients; N-methyl-D-aspartate receptor: 1 patient). We considered them to have “definite autoimmune epilepsy.” The findings are summarized in Table 2. Regarding the remaining four patients, one was classified as having “probable seropositive autoimmune epilepsy” and three were classified as having “possible seropositive autoimmune epilepsy.”

**Table 1:** Demographic clinical data of the patients.

	**Seropositive patients**	**Seroneative patients**	***P*-Value**
Age at onset, median (range)	39 y (9–83)	55 y (14–73)	0.44
Time to admission, median (range)	5.5 mo (1–172)	11 mo (1–420)	0.38
Female sex	11 (79%)	23 (50%)	0.07
History of febrile seizures	0	4 (8.7%)	0.56
Cognitive symtoms	10 (71%)	13 (28%)	0.005
Admission within 6 months (subacute)	8 (57%)	16 (35%)	0.21
AED resistance	12 (86%)	26 (56%)	0.06
Multiple seizure types or FBDS	3 (21%)	0	0.01
Personal history of autoimmunity	4 (29%)	6 (13%)	0.22
Neoplasm or ovarian cyst	0	3 (7%)	1.00
Viral prodrome	1 (7%)	1 (2%)	0.23
Autonomic manifestation	6 (43%)	9 (15%)	0.003

AED, antiepileptic drugs; FBDS, faciobrachial dystonic seizure; mo, month.

**Table 2:** Laboratory data of the patients.

	**Seropositive patients**	**Seronegative patients**	***P*-Value**
CSF abnormality^a^	7/14 (50%)	22/41 (54%)	1.00
MRI abnormality^b^	11 (79%)	24 (52%)	0.12
T2WI/FLAIR HIA in	9 (64%)	16 (34%)	0.07
the temporal lobes			
T2WI/FLAIR HIA in	4 (29%)	4 (8.7%)	0.08
the extratemporal lobes			
Amygdala	5 (36%)	10 (22%)	0.31
enlargement			
FDG-PET hypermetabolism^a^	9/14 (64%)	10/44 (23%)	0.007
Bilateral EEG epileptiform discharge	8 (57%)	15 (32%)	0.12
LGI1	5 (36%)		
GAD	4 (29%)		
NMDAR	1 (7%)		
Seropositive but not specified	4 (29%)		

a Examinations of a limited number of patients. The percentage was calculated based on the results of these patients.

b MRI abnormalities included T2-weighted/FLAIR hyperintense lesions and/or amygdala enlargement.

CSF, cerebrospinal fluid; EEG, electroencephalography; FDG-PET, fluorodeoxyglucose-positron emission tomography; FLAIR, fluid-attenuated inversion recovery; GAD, glutamic acid decarboxylase; HIA, high-intensity area; LGI1, leucine-rich glioma-inactivated 1; MRI, magnetic resonance imaging; NMDAR, N-methyl-D-aspartate receptor.

In terms of classifications according to the diagnostic algorithm among 60 patients, 31 (52%) fulfilled the criteria during the first stage and were considered to have possible autoimmune epilepsy; therefore, they underwent further laboratory evaluations. The remaining 29 patients were classified as having a low possibility of autoimmune epilepsy (group F). During the second stage, 5 patients were classified into group A (8%), 6 were classified into group B (10%), 11 were classified into group C (18%), 7 were classified into group D (12%), and 2 were classified into group E (3%) (Supplementary Figure 1). Among the 14 seropositive patients, 13 proceeded to the second stage and one patient with LGI1-positive antibodies in CSF did not (group F). That one patient had a single generalized tonic-clonic seizure that was controlled by an antiepileptic drug and showed slowly progressive memory impairment and irritability. The EEG results indicated frequent subclinical independent seizures in the bilateral temporal regions, and the MRI and FDG-PET results suggested inflammation of the medial temporal area, including the amygdala, as reported elsewhere (24).

The findings of all patients are summarized in Tables 1, 2, and the clinical and laboratory features of seropositive patients are shown in the Supplementary Table. Abnormalities were observed in seven patients (50%) during the CSF examination, in 11 patients (79%) during the MRI examination, in nine patients (64%) during the FDG-PET examination, and in eight patients (57%) during the EEG examination. Regarding the MRI findings, T2WI/FLAIR high-intensity areas were found in 11 patients; nine were in the temporal lobe and four were in the extratemporal lobe. Amygdala enlargement was observed in five patients. In contrast, three seropositive patients had normal MRI findings.

Regarding MRI abnormalities, 29 patients had temporal lesions, 8 had extratemporal lesions, and 2 had both types of lesions (Table 2). Among the 29 patients with temporal lesions, 25 had T2WI/FLAIR high-intensity lesions and 15 had amygdala enlargement. Eleven patients had both high-signal-intensity lesions and amygdala enlargement. Among the 15 patients with amygdala enlargement, 5 were seropositive and 10 were seronegative. One antibody-positive patient (the aforementioned patient) and six seronegative patients were allocated to group F (24).

**Figure 2:**
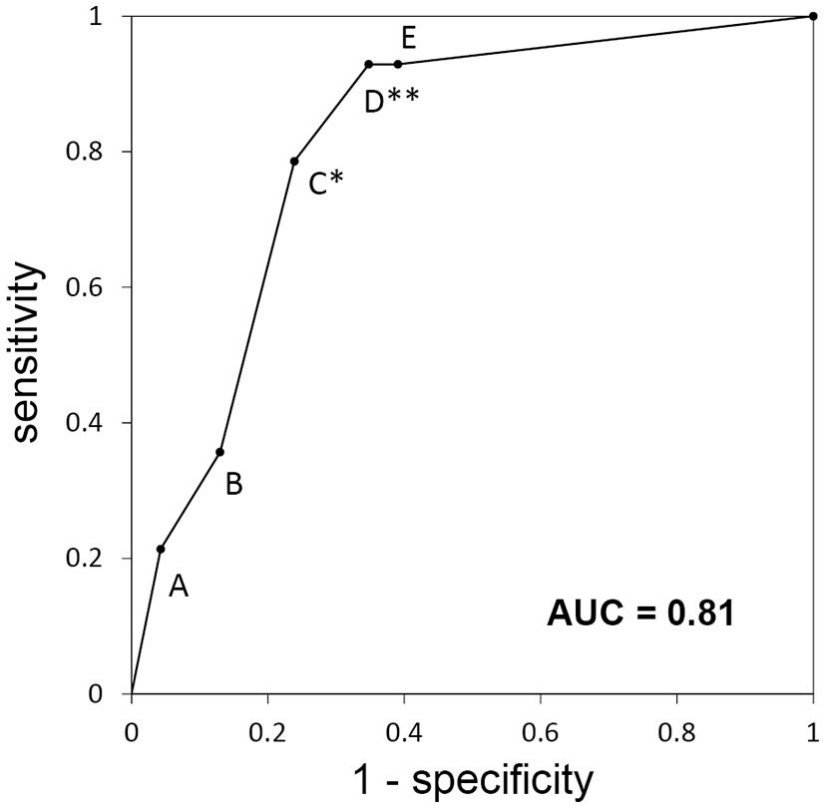
Receiver-operating characteristic curve for the categories of the diagnostic algorithm used for the retrospective cohort. Each alphabet means the sensitivity and specificity when the patients classified A to X are diagnosed with “autoimmune epilepsy.” *Best optimal cutoff; **second optimal cutoff; AUC, area under the curve.

The ROC analysis indicated that the area under the curve of ROC curve was 0.81 (Figure 2) and the most optimal cutoff was identified between groups C and D (sensitivity, 0.79; specificity, 0.76). The second optimal cutoff point was located between groups D and E (sensitivity, 0.93; specificity, 0.65).

A comparison of the diagnostic value of this algorithm with that of the previously published scores (AEP score, ACES score) demonstrated that the APE score showed a sensitivity of 0.93 and specificity of 0.52, and the ACES score showed a sensitivity of 0.86 and a specificity of 0.74.

## Discussion

During this study, we proposed a diagnostic algorithm without antibody testing for autoimmune epilepsy and verified its clinical utility by retrospectively applying it to a cohort who underwent a comprehensive antineuronal antibody work-up. The use of this algorithm showed the possibility of diagnosing autoimmune epilepsy without waiting for the results of antineuronal antibody testing when patients have clinical features suggestive of autoimmune epilepsy and laboratory examination results indicating two or more positive findings (groups A, B, and C). Compared with the preliminary study (11), the revised algorithm had higher sensitivity, and the use of immunohistochemistry improved the accuracy of the diagnosis of autoimmune epilepsy.

The diagnostic criteria for autoimmune limbic encephalitis have been extensively described (8); however, those for autoimmune epilepsy have been rarely reported. To the best of our knowledge, the clinical features suggestive of autoimmune epilepsy and the scoring systems for diagnosing autoimmune epilepsy have been proposed (7, 9, 10). The proposed diagnostic algorithm has two advantages over the scoring systems. First, by using this algorithm, it is possible to follow the actual clinical decision-making process of using clinical features to determine if patients should undergo laboratory examinations. This procedure would help avoid unnecessary examinations (25). Second, the second step of this algorithm incorporates laboratory findings of the CSF, MRI, PET, and EEG examinations more comprehensively. The diagnostic value of EEG findings, that is, bilateral epileptiform discharges, was not evaluated during the two aforementioned studies related to autoimmune epilepsy (8–10). Subclinical seizures detected on EEG (20) or other characteristics, such as dynamic evolution and the involvement of the perisylvian region, have been reported for autoimmune encephalitis, including anti-LGI1 antibody-positive encephalitis (21, 26), indicating the utility of EEG for diagnosing autoimmune epilepsy. Bilateral epileptiform discharges were noted in 39% of the recruited patients. On the other hand, specific EEG findings for autoimmune encephalitis, such as extreme delta brush, were not evaluated in this study. Extreme delta brush is an important EEG finding for anti-NMDAR encephalitis. However, as this is not the case for autoimmune epilepsy, how to handle these EEG findings should be thoroughly discussed.

Although this algorithm focused on the characteristics of autoimmune epilepsy, it did not evaluate psychiatric symptoms or memory impairment, which may occur because of inflammation, especially of the medial temporal structures. Some studies have shown that clinical findings of encephalitis are more associated with antibody positivity than laboratory findings (10, 27). Another report indicated that encephalitis findings are associated with good responsiveness to immunotherapy (27). One LGI1 antibody-positive patient was allocated to group F even though he had both symptoms. This patient fulfilled the diagnostic criteria for definite autoimmune limbic encephalitis but not the criteria of the present diagnostic algorithm or the scoring system for autoimmune epilepsy (8, 9). A forme fruste of limbic encephalitis could manifest as autoimmune epilepsy, but the patient may not be diagnosable by the algorithm/scoring system when the seizure is mild or is not the predominant clinical feature.

The ROC analysis showed that the optimal cutoff point between groups C and D had a sensitivity of 0.79 and a specificity of 0.76. With this cutoff, the algorithm misses approximately one-fifth of autoimmune epilepsy cases. From the viewpoint of treatment, we could choose the second optimal cutoff point between groups D and E (one or more positive findings according to laboratory examinations) that provides higher sensitivity (0.93). This cutoff point is not optimal from a more accurate diagnostic point of view (specificity of 0.65), but it would be beneficial for determining the treatment for autoimmune epilepsy refractive to antiepileptic medications but responsive to first-line immunotherapies, such as intravenous methylprednisolone (28). This is especially true when full antibody testing is unavailable and the patient does not have contraindications for intravenous methylprednisolone.

In our cohort, we included patients who showed positive reactivity for rat brain immunohistochemistry but negative reactivity for antibodies against known or identified antigens, that is, those patients positive for antibodies against unidentified antigens. We defined these patients as having “probable seropositive autoimmune epilepsy” or “possible seropositive autoimmune epilepsy,” depending on the positive results of serum and CSF testing. We should have tested the sera of “possible seropositive autoimmune epilepsy” patients using live neurons to confirm the autoimmune etiology, but it was not feasible to do so because of the limited amount of pretreatment sera available during the present study.

Four patients who had clinical features consistent with autoimmune epilepsy were identified and they underwent evaluations during the second stage. Notably, three patients had examination scores higher than the cutoff point, indicating that they would benefit from immunotherapy. The efficacy of immunotherapy has been reported for patients with suspected autoimmune limbic encephalitis but had negative test results for known antibodies (29). The absence of seizures and improvements in MRI findings, cognition, and mood were observed (14–57%); unidentified antibodies could have been associated with successful immunotherapy.

During this study, 25% of patients tested positive for immunohistochemistry; this value was higher than that reported by previous studies (9, 30). This could be attributed to our hospital being a tertiary epilepsy center where epilepsy specialists are actively diagnosing possible autoimmune epilepsy and performing further evaluations.

Regarding MRI abnormalities, the relationship between amygdala enlargement and epilepsy has recently been highlighted (31, 32). We considered amygdala enlargement as a finding suggestive of autoimmune epilepsy. A total of 15 patients had amygdala enlargement, and five were seropositive. Except for the patient whose predominant clinical features were memory impairment and psychosis (24), all seropositive patients proceeded to the second stage of the algorithm. Regarding the remaining 10 seronegative patients, one patient was allocated to group A, one patient was allocated to group B, one patient was allocated to group C, one patient was allocated to group D, and six patients were allocated to group F. Those in group F may have had pathologies, such as focal cortical dysplasia, hamartomatous lesions, and low-grade glioma, as previously reported (31, 33–35).

When amygdala enlargement occurs in the context of autoimmunity, the enlargement may remit during the clinical course (31). In our cohort, three seropositive but MRI abnormality-negative patients were observed. The amygdala volume of these patients may have been normalized during their clinical course. If available, it is important to obtain previous MRI data, especially when patients have “smoldering” autoimmune epilepsy/limbic encephalitis.

We also included extratemporal lesions as positive MRI finding in our algorithm. Autoimmune encephalitis is often related to limbic encephalitis (9). In our cohort, most patients were considered to have temporal lobe epilepsy with MRI abnormalities in the temporal lobe. However, extratemporal lesions including white matter lesions on MRI are also reported to be frequent in autoimmune epilepsy patients (36). As such, we considered these extratemporal MRI lesions in our algorithm. In this retrospective cohort, extratemporal lesions were observed in eight patients; of these eight patients, four were seropositive. This algorithm successfully detected these seropositive patients. Therefore, it would be more clinically beneficial to include extratemporal abnormalities as positive MRI findings to improve the ability to detect seropositive patients.

When comparing the diagnostic values between this algorithm and previously published scores, the sensitivity and specificity were not very different, taking into account the small size of our cohort. Especially when the second cutoff was used, this algorithm and the APE score showed very similar sensitivity and specificity. It was probably due to the overlap of the factors used in our algorithm and the APE score.

This study had some limitations. First, we applied the proposed algorithm to a retrospective and relatively small cohort and proposed a cutoff value for examinations. The patients were highly selected. In particular, as we included only inpatients, some kind of selection bias could have occurred, including, but not limited to, healthcare access bias (our hospital is a tertiary medical institution for epilepsy) and spectrum bias (the included patients were selected by board-certified neurologists and epileptologists). These facts may have artificially increased the performance of the algorithm. This algorithm should be validated for patients with focal epilepsy and not those with epilepsy with a suspected autoimmune cause. Second, this algorithm involves the potential risk of oversight for patients with other predominant symptoms of autoimmune encephalitis and mild or rare seizures. Whether we should classify these patients as having autoimmune epilepsy should be carefully discussed. Third, anti-NMDA receptor encephalitis is the most common autoimmune encephalitis (37); however, the antibody most frequently detected was the anti-LGI1 antibody and only one patient tested positive for the anti-NMDA receptor antibody during this study. This is in agreement with the finding of a previous study performed at an outpatient clinic (30). Further validation is needed to clarify the antibody that is more associated with autoimmune epilepsy, which is a form of forme fruste of autoimmune encephalitis and may have a smoldering course. Therefore, further immunohistological clarification of unidentified antibodies is also important. Fourth, we evaluated the utility of FDG-PET, but this is only accessible at tertiary epilepsy centers. Single-photon emission computed tomography could be an alternative method because it is more accessible at several hospitals. Some case reports argued the utility of single-photon emission computed tomography hyperperfusion for autoimmune or paraneoplastic encephalitis (38–40). Fifth, anti-MOG antibodies and anti-glycine antibodies are possible causes of autoimmune epilepsy. A broad spectrum of associated clinical phenotypes that can be addressed with anti-MOG antibodies has been shown, and some cases of encephalitis with seizures have been reported (41). In our cohort, we were able to evaluate the sera of only 12 patients for anti-MOG antibodies, and all patients had negative results. This number is too low to evaluate the relevance of MOG antibodies. Anti-glycine antibodies have also been postulated in autoimmune epilepsy (42). However, testing for these antibodies needs to be interpreted carefully, since there are no confirmatory test systems and the syndrome specificity—especially of glycine antibodies—is broad. Nevertheless, anti-MOG and anti-glycine assays should be included in a prospective cohort study in the future. Finally, validation of the cutoff value and modification of the algorithm using a larger prospective cohort are warranted to establish the diagnostic algorithm for autoimmune epilepsy.

## Conclusion

We proposed a diagnostic algorithm without antibody testing for autoimmune epilepsy that followed real-world practical procedures ranging from assessing the medical history/clinical symptoms to performing laboratory examinations. We verified its clinical utility by retrospectively applying the algorithm to a cohort who underwent a comprehensive antineuronal antibody work-up. This study provides Class IV evidence that this algorithm improves the diagnostic accuracy when autoimmune epilepsy is suspected, when patients have clinical features suggestive of autoimmune epilepsy, and when patients have two or more positive laboratory examination results.

## Data availability statement

The data that support the findings of this study are available on request from the corresponding authors (RM and AI). The data are not publicly available because they contain information that could compromise the privacy of the research participants.

## Ethics statement

The studies involving human participants were reviewed and approved by Kyoto University Graduate School and Faculty of Medicine, Ethics Committee. Written informed consent to participate in this study was provided by the participants and/or their legal guardian/next of kin.

## Author contributions

Conceptualization was performed by RM, TK, RT, and AI. Methodology was created by MS, RM, FL, K-PW, TK, and AI. Formal analysis and investigation were performed by MS, RM, FL, and K-PW. Resources were arranged by RM, JT, HT, KK, AS, FL, K-PW, and AI. The manuscript was written by MS and reviewed and edited by RM, AS, FL, and AI. RM, FL, K-PW, and AI were responsible for funding acquisition. RT and AI supervised this research. All authors contributed to the article and approved the submitted version.

## Funding

This work was partly supported by JSPS KAKENHI (15H05874), a research grant from the Japan Epilepsy Research Foundation (JERF TENKAN 20010), and the German Ministry of Education and Research (BMBF, 01GM1908A, Leypoldt).

## Conflict of interest

Department of Epilepsy, Movement Disorders, and Physiology was an endowment department supported by a grant from GlaxoSmithKline K.K., NIHON KOHDEN CORPORATION, Otsuka Pharmaceutical Co., and UCB Japan Co., Ltd. until May 2018. Since 1 June 2018, this department has changed to Industry-Academia Collaboration Courses supported by a grant from Eisai Co., Ltd., NIHON KOHDEN CORPORATION, Otsuka Pharmaceutical Co., and UCB Japan Co., Ltd. Author AI is a current member of this department, and Authors RM and AS are previous members of this department. Department of Respiratory Care and Sleep Control Medicine is funded by endowments from Philips Japan, ResMed, Fukuda Denshi, and Fukuda Lifetec Keiji to Kyoto University. Author JT is a current member of this department, and author HT is a previous member of this department. Authors K-PW and FL report speakers honoraria from Bayer, Roche, Novartis, and Fresenius, have received travel funding from Merck, Grifols, and Bayer, and serve on the advisory boards for Roche, Biogen, and Alexion. The remaining authors declare that the research was conducted in the absence of any commercial or financial relationships that could be construed as a potential conflict of interest.

## Publisher's note

All claims expressed in this article are solely those of the authors and do not necessarily represent those of their affiliated organizations, or those of the publisher, the editors and the reviewers. Any product that may be evaluated in this article, or claim that may be made by its manufacturer, is not guaranteed or endorsed by the publisher.
