# Supplementary material for: Diagnostic value of an algorithm for autoimmune epilepsy in a retrospective cohort
Source: Front Neurol. 2022 Sep 14;13:902157. doi: 10.3389/fneur.2022.902157 (PMC9518792; doi:10.3389/fneur.2022.902157)
Supplement: Supplementary file 1 [file Data_Sheet_1.pdf]

Supplementary Material

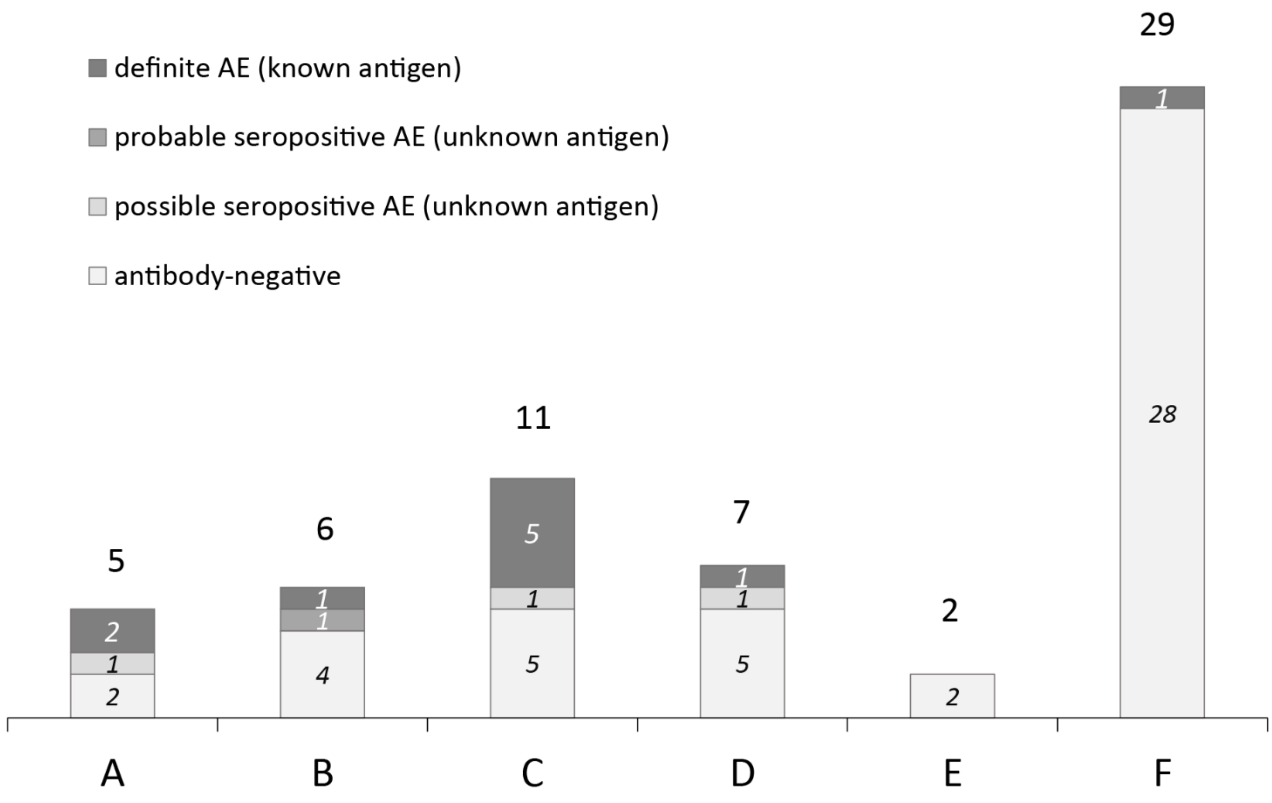

Supplementary Figure 1. Bar graph of the number of patients in each category

Patients in each category (A–F) of the proposed algorithm. The darkest gray bar denotes patients with positive immunohistochemistry results and positive results for specific antigens (definite autoimmune epilepsy). The second darkest gray bar denotes those with positive CSF in rat brain immunohistochemistry results without detectable known antigens (probable seropositive autoimmune epilepsy). The third darkest gray bar denotes those with positive rat brain immunohistochemistry serum sample results but negative CSF sample results or unavailable CSF samples (possible seropositive autoimmune epilepsy). The light gray bar denotes those with negative rat brain immunohistochemistry. The number in each bar denotes the number of patients.

AE, autoimmune epilepsy; CSF, cerebrospinal fluid

Supplementary Table 1-1. The clinical features of the seropositive patients

Pt. No	age at onset	Duration [a] (m)	sex	Antibody	epilepsy type	seizure type [b]	History of FS	Behavioral change	Cognitive symptoms	Autoimmune disease	autonomic manifestation
#1	59	5	F	LGI1 (C)	TLE	CPS, FBDS	none	irritability	none	none	goose bumps, water drinking
#2	62	8	M	LGI1 (C)	TLE	SPS, GTCS	none	irritability	memory impairment	none	none
#3	42	25	F	unknown (S)	Lt. TLE	SPS, CPS, GTCS	none	irritability	memory impairment	none	goose bumps
#4	16	5	F	GAD (C)	TLE	SPS, GTCS	none	none	memory impairment	none	(dizziness)
#5	65	3	F	LGI1 (C)	TLE	SPS	ND	depression, sexual deviance	memory impairment	none	none
#6	36	6	M	LGI1 (C, S)	Lt. FLE	SPS, GTCS, FBDS	none	none	none	none	goose bumps
#7	9	172	F	GAD (C)	TLE	CPS, GTCS	none	none	low IQ	DM type 1	(nausea)
#8	62	2	F	LGI1 (C)	focal epilepsy	CPS, SPS, GTCS	none	abnormal behavior	memory impairment	none	none
#9	30	144	F	unknown (S)	Rt. TLE	SPS, CPS	ND	suicide attempt	memory impairment	none	hyperventilation, water drinking
#10	28	2	F	GAD (C)	TLE	GTCS, CPS	none	none	memory impairment	Hashimoto disease	spitting
#11	36	49	F	GAD (C)	TLE	GTCS, CPS	none	none	memory impairment	DM type 1	none
#12	59	8	F	unknown (S)	TLE	GTCS, CPS, SPS	none	none	none	none	tachycardia
#13	83	1	F	unknown (C)	focal epilepsy	CPS, NCSE	ND	none	Disturbance of consciousness	RA	none
#14	26	1	M	NMDAR (S)	focal epilepsy	GTCS	ND	abnormal behavior, agitation	none	none	none

C, cerebrospinal fluid; CPS, complex partial seizure; DM, diabetes mellitus; F, female; FBDS, faciobrachial dystonic seizure; FS, febrile seizure; GAD, glutamic acid decarboxylase; GTCS, generalized tonic-clonic seizure; IQ, intelligence quotient; LGI1, leucine-rich glioma-inactivated 1; Lt, left; M, male; m, month; NCSE, non-convulsive status epilepticus; ND, no data; NMDAR, N-methyl-D-aspartate receptor; RA, rheumatoid arthritis; Rt, right; S, serum; SPS, simple partial seizure; TLE, temporal lobe epilepsy.

[a] This means the period form the seizure onset to hospitalization for evaluation and treatment.

[b] These terms are used based on the medical chart because patients were recruited from January 2012 to March 2017

Supplementary Table 1-2. The laboratory findings and other features of the seropositive patients

Pt. No	AED	CSF findings	MRI findings	FDG-PET findings	EEG findings			classification of algorithm
					epileptiform discharge	onset of seizure pattern	others	
#1	VPA, LEV, CBZ	normal	Lt.T HIA, AE	Lt.T hyper	none	none	Lt.T slow	C
#2	VPA	normal	Bil.T HIA, AE	Bil.T hyper	Lt.FT (change to Bil.FT)	Bil.T	Bil.FT slow	F
#3	VPA, ZNS	protein ↑	Bil.mT HIA, AE	Lt.T hyper	Lt.FT (change to Rt.FT)	none	Rt.T slow	A
#4	LEV, TPM	OCB (+), protein↑	normal	Bil.T hypo	Bi.FT	Bil.T	Bi.FT slow	C
#5	VPA	normal	Lt.mT HIA, AE	Lt.T hyper	none	none	Lt.FT slow	C
#6	VPA, LEV, CLB	normal	Lt.F HIA	Lt.F hyper	none	Rt.FT	Rt.F slow, Lt.C slow	C
#7	CBZ, VPA, PB	normal	normal	normal	Bil.T	not available	Bil.T slow	D
#8	PHT, CZP, LEV, TPM, LTG	protein ↑	Bil.T HIA	Rt.T hypo	none	Lt.T	Lt.post T slow	C
#9	VPA, CBZ, TPM	normal	normal	normal	Lt. posterior T	Bil.T	Bil.FT slow	D
#10	CBZ, LEV, CLB	OCB (+)	Bil.T+Insula HIA, AE	Bil.T hyper	Rt.FT (change to Bil.FT)	Bil.T	Bil.T slow	A
#11	VPA, CBZ	protein ↑	Lt.HS	Lt.T hyper	Bil.AT	Bil.T	Bil.AT slow	A
#12	LEV, CLB	normal	Lt.T white matter HIA	normal	Bil.basal T	Lt.T	Bil.basal T slow	C
#13	LCM, LEV	protein ↑	multiple HIA	multiple hyper	none	Lt.post T periodic pattern	Lt.post T slow	B
#14	PHT, propofol, MDZ	cell ↑	multiple HIA	Rt hemisphare hyper	none	not available	Extreme delta brush	B

AE, amygdala enlargement; AED, antiepileptic drugs; Bil, bilateral; C, central; CBZ, carbamazepine; CLB, clobazam; CSF, cerebrospinal fluid; CZP, clonazepam; EEG, electroencephalogram; FDG-PET, fluorodeoxyglucose-positron emission tomography ; FT, frontotemporal; HIA, high intensity area; HS, hippocampal sclerosis; Lt, left; hyper, hypermetabolism; hypo, hypometabolism; LCM, lacosamide; LEV, levetiracetam; MDZ, midazolam; MRI, magnetic resonance imaging; OCB, oligoclonal band; PB, phenobarbital; PHT, phenytoin; post, posterior; Rt, right; T, temporal; TPM, topiramate; VPA, valproic acid; ZNS, zonisamide; ↑, elevated protein level or elevated cell count.
